# Isolation and Characterization of a Naturally Occurring *Brevundimonas vesicularis* Strain Exhibiting High Phytoene Accumulation

**DOI:** 10.3390/foods15172981

**Published:** 2026-08-25

**Authors:** Zhenyi Liu, Ying Liu, Yan Zhi, Chen Mei, Hongjun Wang

**Affiliations:** 1 Institute of Animal Husbandry and Veterinary Medicine, Beijing Academy of Agriculture and Forestry Sciences, Beijing 100097, Chinaliuying@baafs.net.cn (Y.L.);; 2 College of Veterinary Medicine, China Agricultural University, Beijing 100193, China

**Keywords:** phytoene, *Brevundimonas vesicularis*, carotenoids, microbial fermentation, functional food ingredients

## Abstract

Phytoene, a colorless precursor of carotenoids, has attracted increasing attention because of its favorable bioavailability, antioxidant activity, and potential applications in functional foods, nutraceuticals, and animal nutrition. However, its industrial utilization remains limited by low natural abundance and the dependence of current production strategies on genetic engineering or metabolic pathway manipulation. In this study, we identified and characterized a naturally occurring *Brevundimonas vesicularis* strain (Bv-xms2024) exhibiting pronounced phytoene accumulation without genetic modification. The strain was comprehensively characterized using morphological, biochemical, molecular, genomic, metabolomic, and transcriptional analyses. Quantitative LC–MS/MS analysis demonstrated that Bv-xms2024 accumulated phytoene to 420.42 ± 98.11 μg/g dry biomass after 96 h of cultivation, substantially exceeding the levels of downstream carotenoids, including β-carotene and astaxanthin. Optimization of cultivation parameters identified 25 °C, pH 7.0, and 96 h as the optimal conditions for phytoene accumulation, while serial passaging confirmed stable production over 20 generations. Genome annotation identified the carotenoid biosynthetic gene repertoire, while RT-qPCR analysis revealed a temporal shift from early upregulation of *crtE* and *crtB* to later upregulation of downstream pathway genes, consistent with the observed phytoene-dominant carotenoid profile. Short-term tolerance evaluations in mice and chickens revealed no observable adverse effects under the tested conditions. Collectively, these findings identify Bv-xms2024 as a promising natural microbial resource for phytoene production and provide a basis for further process development and strain-level safety evaluation.

## 1. Introduction

Carotenoids are naturally occurring isoprenoid pigments widely distributed in plants, algae, fungi, and diverse bacteria, where they play essential roles in light harvesting, photoprotection, and oxidative stress defense [1]. Among them, phytoene is the first committed colorless intermediate in the carotenoid biosynthetic pathway and serves as the precursor for the synthesis of colored carotenoids such as lycopene, β-carotene, and astaxanthin [2]. Unlike highly pigmented carotenoids, phytoene exhibits favorable bioavailability [3,4] and has demonstrated antioxidant and anti-inflammatory activities [5,6], making it an attractive candidate for applications in functional foods, nutraceuticals, and cosmetics [3,7], as well as animal nutrition [8]. Increasing evidence suggests that dietary phytoene may contribute to protection against oxidative stress and UV-induced skin damage [6,7], highlighting its growing commercial and nutritional value.

Despite its considerable biological potential, commercial production of phytoene remains challenging because of its naturally low abundance in most biological sources and its rapid metabolic conversion into downstream carotenoids [9]. Current production strategies mainly rely on metabolic engineering, pathway inhibition, or heterologous expression systems to redirect metabolic flux toward phytoene accumulation [9,10]. Although these approaches have substantially improved production yields, their industrial translation still requires cost-effective downstream processing and may face regulatory constraints for food-related applications [11,12]. Consequently, the discovery of naturally occurring microorganisms capable of accumulating phytoene without genetic manipulation represents a promising complementary strategy for future industrial production.

Microbial fermentation has emerged as an efficient platform for the potentially sustainable production of natural food ingredients because of its short production cycle, controllable cultivation conditions, and scalability [13]. Although numerous microorganisms have been investigated as carotenoid producers, current microbial research and biomanufacturing efforts remain concentrated on β-carotene, lycopene, astaxanthin, and other colored carotenoids, whereas naturally occurring microorganisms characterized by substantial phytoene accumulation remain comparatively underexplored [2,13]. Therefore, identifying naturally occurring phytoene-producing microorganisms remains important for expanding the microbial resources available for food biotechnology.

Several members of the genus *Brevundimonas* have been reported to possess carotenoid biosynthetic gene clusters and to synthesize structurally diverse xanthophylls, including hydroxylated astaxanthin derivatives [14,15]. Nevertheless, previous studies have mainly focused on the functional characterization of carotenoid biosynthetic genes and downstream xanthophyll formation, whereas phytoene accumulation in naturally occurring *Brevundimonas* strains remains poorly characterized [14,15]. Given that some *Brevundimonas* species, including *B. vesicularis*, have been recognized as opportunistic pathogens, strain-level genomic screening and safety assessment are particularly important for their potential food-related development [16]. Moreover, comprehensive evaluations integrating genome sequencing, carotenoid metabolomics, transcriptional analysis, production stability, and biological safety are still lacking. These knowledge gaps have limited the exploration of *Brevundimonas* as a potential microbial resource for natural carotenoid production.

In the present study, we isolated and characterized a naturally occurring *Brevundimonas vesicularis* strain, designated Bv-xms2024, that exhibited pronounced phytoene accumulation. The strain was systematically evaluated through phenotypic and molecular characterization, whole-genome sequencing, quantitative carotenoid profiling, transcriptional analysis of key carotenoid biosynthetic genes, optimization of cultivation conditions, assessment of production stability, and preliminary safety evaluation in mice and chickens. This study aimed to assess the potential of Bv-xms2024 as a natural microbial source of phytoene and to provide a foundation for further process development and comprehensive safety assessment for food- and feed-related applications.

## 2. Materials and Methods

### 2.1. Ethics Approval and Consent to Participate

All animal experiments were approved by the Animal Care and Use Committee of the Institute of Animal Husbandry and Veterinary Medicine, Beijing Academy of Agriculture and Forestry Sciences (protocol code: IHVM11-2409-77, date of approval: 20 August 2024). The mouse and chicken experiments were conducted in accordance with the institutional guidelines for animal care and use. All efforts were made to minimize animal suffering.

### 2.2. Media and Culture Conditions

Luria–Bertani (LB) broth and LB agar (Beijing Land Bridge Technology Co., Ltd., Beijing, China) were used for bacterial isolation, cultivation, and routine maintenance. Unless otherwise specified, cultures were incubated aerobically at 25 °C with shaking at 170 rpm, while agar plates were incubated statically at the same temperature. All chemicals used in this study were of analytical grade, and ultrapure water was used throughout the experiments.

### 2.3. Isolation and Purification of Strain Bv-xms2024

Strain Bv-xms2024 was isolated in 2024 from an environmental sample during routine microbial culture. Orange-red pigmented colonies were observed on LB agar after incubation at 25 °C. Individual colonies were repeatedly purified by successive streaking until a morphologically homogeneous isolate was obtained. The purified isolate was designated Bv-xms2024 and maintained on LB agar for subsequent experiments.

### 2.4. Morphological Characterization

Colony morphology was examined after 24 h of incubation on LB agar at 25 °C. Single colonies were picked with a sterile inoculating loop and suspended in distilled water on glass slides. Gram staining was performed using a commercial Gram staining kit according to the manufacturer’s instructions (Beijing Solarbio Science & Technology Co., Ltd., Beijing, China), and bacterial morphology was observed under a light microscope (Nikon, Tokyo, Japan).

Physiological and biochemical tests were conducted following the protocols outlined in Bergey’s Manual of Systematic Bacteriology and the Manual of Common Bacterial Identification. Tests included catalase activity, oxidase activity, citrate utilization, indole production, urease activity, hydrogen sulfide production, and carbohydrate fermentation profiles using glucose, lactose, and sucrose [17,18]. All reagents and media were obtained from standard suppliers (Hopebio Co., Ltd., Qingdao, China).

### 2.5. Molecular Identification

A single colony of Bv-xms2024 was inoculated into LB broth and cultured at 25 °C with shaking at 170 rpm for 24 h. Genomic DNA was extracted using a TIANamp Bacteria DNA Kit (Tiangen Biotech Co., Ltd., Beijing, China) according to the manufacturer’s instructions.

The 16S rRNA gene was amplified using the universal primers 27F (5′-AGAGTTTGATCCTGGCTCAG-3′) and 1492R (5′-TACGGCTACCTTGTTACGACTT-3′), which were synthesized by BGI Tech Solutions Co., Ltd. (Beijing, China). PCR amplification was performed in a total volume of 50 μL containing 2 μL of forward primer (10 μM), 2 μL of reverse primer (10 μM), 2 μL of DNA template, 25 μL of 2× Taq Master Mix (Vazyme Biotech Co., Ltd., Nanjing, China), and 19 μL of nuclease-free water. The PCR conditions were as follows: initial denaturation at 94 °C for 4 min; 30 cycles of denaturation at 94 °C for 45 s, annealing at 56 °C for 30 s, and extension at 72 °C for 2 min; and final extension at 72 °C for 10 min. Negative controls containing nuclease-free water instead of DNA template were included in each PCR run.

PCR products were analyzed by electrophoresis on a 1.0% (*w*/*v*) agarose gel stained with GelRed nucleic acid stain (Beijing Biomed Gene Technology Co., Ltd., Beijing, China) and visualized under UV light using a Gel Doc XR+ system (Bio-Rad Laboratories, Hercules, CA, USA). The amplified products were purified using a QIAquick PCR Purification Kit (Qiagen, Hilden, Germany) and sent to BGI Tech Solutions Co., Ltd. for Sanger sequencing.

The obtained 16S rRNA gene sequence was compared with sequences in the National Center for Biotechnology Information (NCBI) GenBank database using BLASTN. Multiple sequence alignments were performed using ClustalW in MEGA 11 [19]. A phylogenetic tree was constructed using the neighbor-joining method with 1000 bootstrap replicates.

### 2.6. Determination of the Bacterial Growth Curve

To determine the growth kinetics of Bv-xms2024, representative colonies were selected from LB agar plates and inoculated into LB broth. Cultures were incubated at 25 °C with shaking at 170 rpm for 24 h. The resulting seed culture was adjusted to an optical density at 600 nm (OD_600_) of 0.30 using a BioPhotometer Plus spectrophotometer (Eppendorf, Hamburg, Germany). A 2% (*v*/*v*) inoculum was then transferred into 400 mL of fresh LB broth and incubated under the same conditions.

Culture samples were collected aseptically at 0 h and then every 2 h until 72 h. Samples were serially diluted 10-fold in sterile phosphate-buffered saline (PBS), spread onto LB agar plates, and incubated at 25 °C for 72 h. Colony-forming units (CFUs) were counted, and viable counts were expressed as log_10_(CFU/mL). Growth parameters, including specific growth rate, generation time, and decay half-life, were calculated from semi-logarithmic growth curves as described previously [20].

### 2.7. Antibiotic Susceptibility Testing

The antibiotic susceptibility of strain Bv-xms2024 was determined using the disk diffusion method on Mueller–Hinton (MH) agar plates, following the guidelines of the Clinical and Laboratory Standards Institute (CLSI) [21]. The bacterial suspension was prepared by adjusting the turbidity of an overnight culture (24 h at 25 °C in LB broth) to match a 0.5 McFarland standard (approximately 1.5 × 10^8^ CFU/mL).

Sterile cotton swabs were dipped into the standardized bacterial suspension, excess liquid was removed by pressing against the inner wall of the tube, and the swab was used to evenly inoculate the entire surface of the MH agar plates by swabbing in three directions. Twenty-three antibiotics representing β-lactams, aminoglycosides, quinolones, macrolides, tetracyclines, sulfonamides, lincosamides, polypeptides, and phenicols were evaluated. After allowing the antibiotic disks (Liofilchem S.r.l., Roseto degli Abruzzi, Italy) to adhere and the bacterial suspension to absorb into the agar (approximately 15 min), the plates were inverted and incubated at 25 °C for 24–48 h. The inhibition zones were observed, and the diameters were measured to the nearest millimeter using a caliper. Each test was performed in triplicate, and the average inhibition zone diameters were calculated. Because no CLSI interpretive criteria are available for *B. vesicularis*, inhibition zone diameters were reported descriptively without assigning susceptible, intermediate, or resistant categories.

### 2.8. Carotenoid Extraction and Analysis

Representative colonies were selected from LB agar plates and inoculated into LB broth. The cultures were incubated at 25 °C with shaking at 170 rpm for 96 h. Cell-pellet samples were freeze-dried, ground at 30 Hz for 1 min, and 50 mg of the resulting powder was extracted twice with n-hexane/acetone/ethanol (1:1:1, *v*/*v*/*v*) containing 0.01% butylated hydroxytoluene (BHT). The combined extracts were concentrated, reconstituted in 150 μL dichloromethane, filtered through a 0.22 μm membrane, and subjected to LC–MS/MS analysis. Detailed sample-preparation procedures are provided in Supplementary Method S1.

Carotenoids were analyzed using an ExionLC™ AD UPLC system coupled to a QTRAP^®^ 6500+ mass spectrometer (SCIEX, Framingham, MA, USA) equipped with an APCI source. Separation was performed on a YMC C30 column (YMC Co., Ltd., Kyoto, Japan) (3 μm, 100 × 2.0 mm) using methanol/acetonitrile and methyl tert-butyl ether-based mobile phases, followed by scheduled multiple reaction monitoring (MRM). Quantification was performed against external-standard calibration curves using MultiQuant 3.0.3 (SCIEX, Framingham, MA, USA). Full chromatographic conditions, MS/MS parameters, standard preparation, and calibration procedures are provided in Supplementary Method S1. Carotenoid contents are expressed as μg/g dry biomass.

### 2.9. Optimization of Cultivation Parameters, Orthogonal Experimental Design and Stability Assessment

To evaluate the effects of basic cultivation parameters on phytoene accumulation, cultivation time, temperature, and initial pH were investigated in shake-flask cultures. Briefly, strain Bv-xms2024 was inoculated into LB broth at 2% (*v*/*v*) and cultivated aerobically at 170 rpm. Temperature was set at 20, 25, or 30 °C, and the initial pH was adjusted to 6.5, 7.0, or 7.5 by adding 1.0 M HCl or 1.0 M NaOH.

An L9 (3^3^) orthogonal array was then used to examine the combined effects of cultivation time (48, 72, and 96 h), temperature (20, 25, and 30 °C), and initial pH (6.5, 7.0, and 7.5) and to identify an improved parameter set. The experimental matrix is shown in Table 1. For each run, cultures were harvested at the predefined time point for phytoene quantification. Phytoene was determined using the same carotenoid extraction procedure and LC–MS/MS workflow described above. The initial carotenoid-profiling and cultivation-optimization experiments were conducted independently using the same extraction and analytical procedures. All runs were performed in triplicate (n = 3).

Production stability during serial passaging. To evaluate multi-generation stability, strain Bv-xms2024 was serially passaged to generation 20 (G20). Phytoene accumulation was assessed at G1, G10, and G20 under the selected cultivation condition (25 °C, initial pH 7.0, 96 h) using the same extraction procedure and LC–MS/MS workflow described above. Three independent biological replicates were analyzed for each generation (n = 3).

### 2.10. Whole-Genome Sequencing, Assembly, and Functional Annotation

Genomic DNA of strain Bv-xms2024 was sequenced using the Oxford Nanopore platform by Jiangsu Cowin Biotech Co., Ltd. (Taizhou, Jiangsu, China). Raw reads were quality-checked and filtered to obtain clean reads. Long-read assembly was performed and the consensus sequence was polished. Clean reads were mapped back to the assembly to assess genome coverage and sequencing depth, and circularity was evaluated based on the assembly structure and mapping consistency. A taxonomic screen was conducted to confirm that the dataset was dominated by *Brevundimonas* sequences.

The final assembly was structurally annotated to generate standard annotation files (GFF3/GenBank/EMBL) and protein/nucleotide FASTA outputs. Functional annotation was performed using Non-redundant protein database (NR)/Swiss-Prot, Clusters of Orthologous Groups (COG), Kyoto Encyclopedia of Genes and Genomes (KEGG), Gene Ontology (GO), and Carbohydrate-Active enZymes database (CAZy), and additional database screening was carried out using Comprehensive Antibiotic Resistance Database (CARD), Virulence Factor Database (VFDB), and Pathogen–Host Interactions database (PHI).

### 2.11. Validation of Key Carotenoid Biosynthetic Genes by Reverse Transcription-Quantitative PCR (RT-qPCR)

Total RNA was extracted from the cultures at 24, 48, 72, and 96 h using an RNA extraction kit (TRIzol reagent, Invitrogen, Carlsbad, CA, USA), followed by complementary DNA (cDNA) synthesis using TransScript^®^ II First-Strand cDNA Synthesis SuperMix (TransGen Biotech Co., Ltd., Beijing, China). The key genes involved in the carotenoid biosynthesis pathway, including *crtY*, *crtW*, *crtI*, *crtB*, *crtE*, and *crtZ*, were quantified using quantitative PCR (qPCR) with the primer sequences shown in Table 2.

RT-qPCR was performed using the 2× Hieff UNICON^®^ Universal Blue qPCR Master Mix (Yeasen Biotech Co., Ltd., Shanghai, China) under the following amplification con-ditions: activation of Uracil-DNA Glycosylase (UDG) at 50 °C for 2 min, initial denaturation at 95 °C for 2 min, followed by 40 cycles of denaturation at 95 °C for 15 s and annealing/extension at 60 °C for 30 s, concluding with melting curve analysis. All experiments were conducted in triplicate. The 16S rRNA gene served as the internal control, and gene expression fold changes were calculated using the 2^−ΔΔCT^ method, with PCR efficiency confirmed to be consistent across all targets [22].

### 2.12. Animal Safety Evaluation

This section presents the 14-day animal tolerance evaluations of Bv-xms2024 in mice and chickens.

#### 2.12.1. Mouse Safety Evaluation

Forty healthy Kunming mice with similar body weights were randomly divided into four groups of ten mice in each group (five males and five females). The control group received 0.2 mL of sterile saline via oral gavage, whereas the other three groups received 0.2 mL of the bacterial suspension of strain Bv-xms2024 at concentrations of 1 × 10^9^ CFU/mL (LG group), 1 × 10^10^ CFU/mL (MG group), and 1 × 10^11^ CFU/mL (HG group). The experimental duration was 14 days, during which time the mice were weighed every 2 days and their health status was recorded.

#### 2.12.2. Chicken Safety Evaluation

To assess the tolerance of strain Bv-xms2024, 40 chickens were used, including 20 specific-pathogen-free (SPF) chicks aged 42 days and 20 laying hens aged 270 days. For each age cohort, the chickens were divided into a blank control group and an experimental group, with ten chickens per group. Before the experiment, the chickens underwent a 1-week acclimatization period. The animals were housed in separate SPF units with ad libitum access to feed and water. For preparation of the bacterial culture used for exposure, the seed culture was adjusted to an OD600 of 0.5 and inoculated into fresh LB medium at a ratio of 1:100 (*v*/*v*), followed by shaking cultivation for 96 h. Viable bacterial counts were determined for each preparation to confirm the consistency of bacterial loads among parallel cultures. The blank control groups received normal drinking water, whereas the experimental groups received the 96-h Bv-xms2024 culture ad libitum as the drinking source. The bacterial drinking culture was replaced every 48 h. Because the culture was administered through ad libitum drinking, a fixed administration volume and exact bacterial intake per individual bird were not predetermined. The same standardized culture-preparation and exposure procedure was applied to both age cohorts. The experimental period lasted for 14 days, during which time the body weights of the chickens were recorded every 2 days along with their health status.

### 2.13. Statistical Analysis

Statistical analyses were conducted using GraphPad Prism (Version 8.0, San Diego, CA, USA), with results presented as the mean ± standard deviation (SD). Differences between two groups were evaluated using an unpaired Student’s *t*-test, while comparisons among multiple groups were performed using one-way analysis of variance (ANOVA) followed by Tukey’s post hoc test. For the RT-qPCR analysis, a relative quantification method (2^−ΔΔCT^) was employed. A *p*-value < 0.05 was considered statistically significant.

## 3. Results

### 3.1. Morphological Characteristics of the Strain

**Figure 1:**
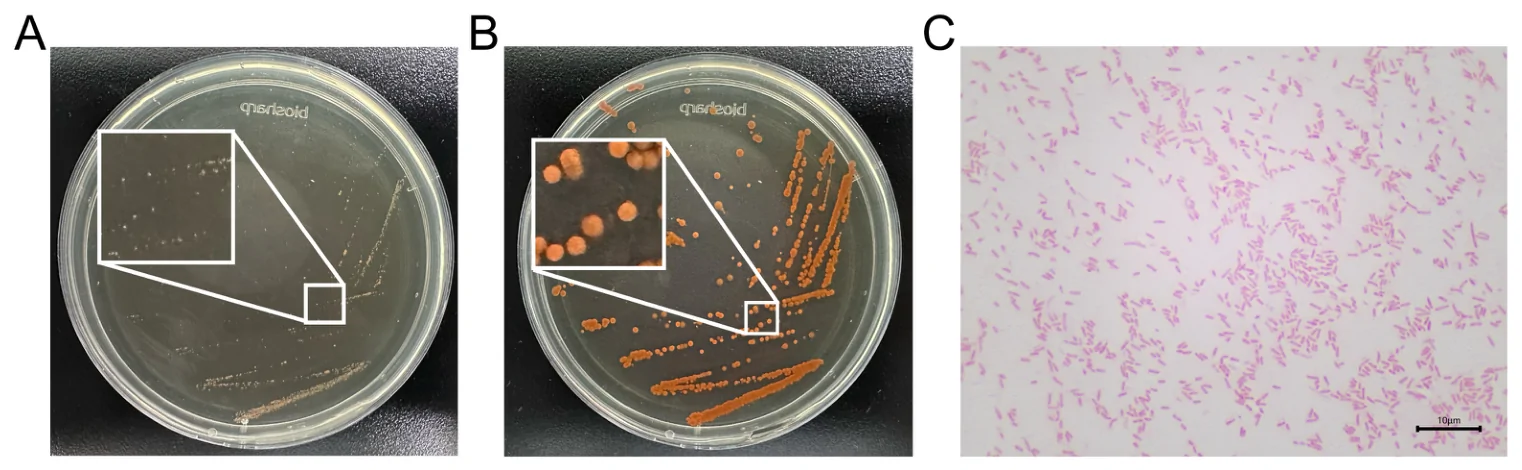
Morphological characteristics of strain Bv-xms2024. (**A**) Appearance of Bv-xms2024 colonies on Luria–Bertani (LB) agar before 24 h of incubation. (**B**) Appearance of Bv-xms2024 colonies on LB agar after 24 h of incubation, showing orange-yellow pigmentation. (**C**) Microscopic image of Bv-xms2024 cells after Gram staining.

On LB agar medium, colonies of Bv-xms2024 exhibited distinct morphological changes over time. Prior to 24 h of incubation, the colonies appeared as shown in Figure 1A; they were circular with smooth margins, centrally raised, and exhibited a transparent to slightly whitish coloration. After 24 h of incubation, the appearance of the colonies changed, as depicted in Figure 1B. They remained circular with smooth edges and centrally raised, but developed orange-yellow pigmentation. The intensity of this coloration deepened progressively with extended incubation time.

Microscopic examination of the bacterial cells (Figure 1C) revealed that they were short rods. Gram staining indicated that the bacteria stained red, indicating that strain Bv-xms2024 was Gram-negative.

### 3.2. Physiological and Biochemical Characteristics

The physiological and biochemical characteristics of strain Bv-xms2024 are summarized in Table 3. The strain was positive for citrate utilization, indicating its ability to use citrate as a sole carbon source. Additionally, the strain exhibited catalase activity but was oxidase-negative. It did not produce hydrogen sulfide and was negative for indole production and urease activity. Carbohydrate fermentation tests indicated that the strain could ferment glucose and sucrose but not lactose.

### 3.3. Molecular Identification and Phylogenetic Analysis

The 16S rRNA gene of Bv-xms2024 was successfully amplified by PCR, yielding a product of approximately 1500 bp. BLASTN analysis of the 16S rRNA gene sequence showed 99.5% similarity to *Brevundimonas vesicularis* reference sequences.

**Figure 2:**
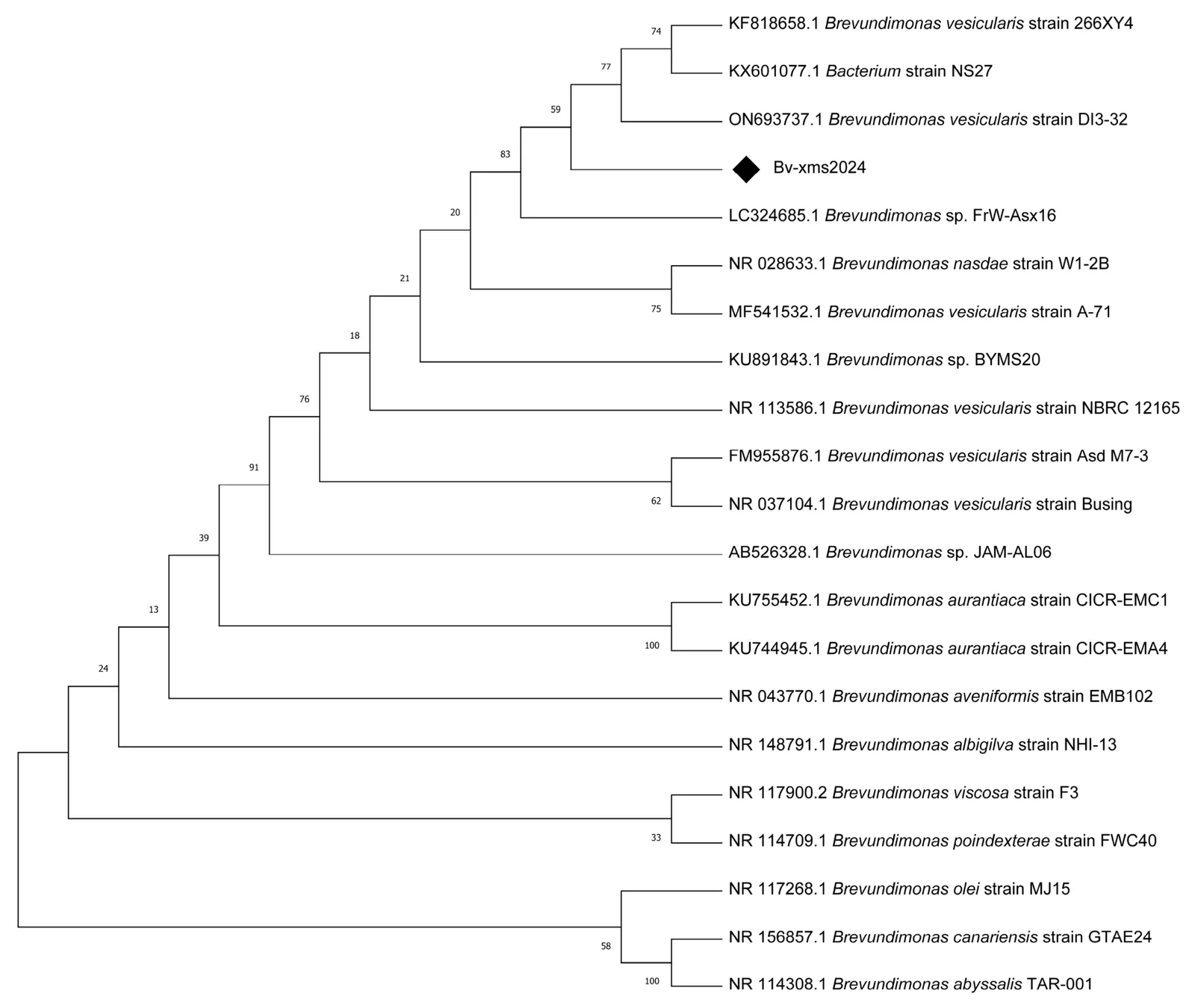
Phylogenetic analysis of strain Bv-xms2024 based on 16S rRNA gene sequences. The tree was constructed using the neighbor-joining method in MEGA 11. Bv-xms2024 is indicated by a black diamond. Numbers at branch nodes indicate bootstrap values.

A phylogenetic tree constructed using the neighbor-joining method in MEGA 11 showed that Bv-xms2024 clustered within the *Brevundimonas* clade and was closely related to *B. vesicularis* strains (Figure 2). Based on the morphological, biochemical, and molecular evidence, the strain was designated *Brevundimonas vesicularis* Bv-xms2024.

### 3.4. Growth Curve Analysis

**Figure 3:**
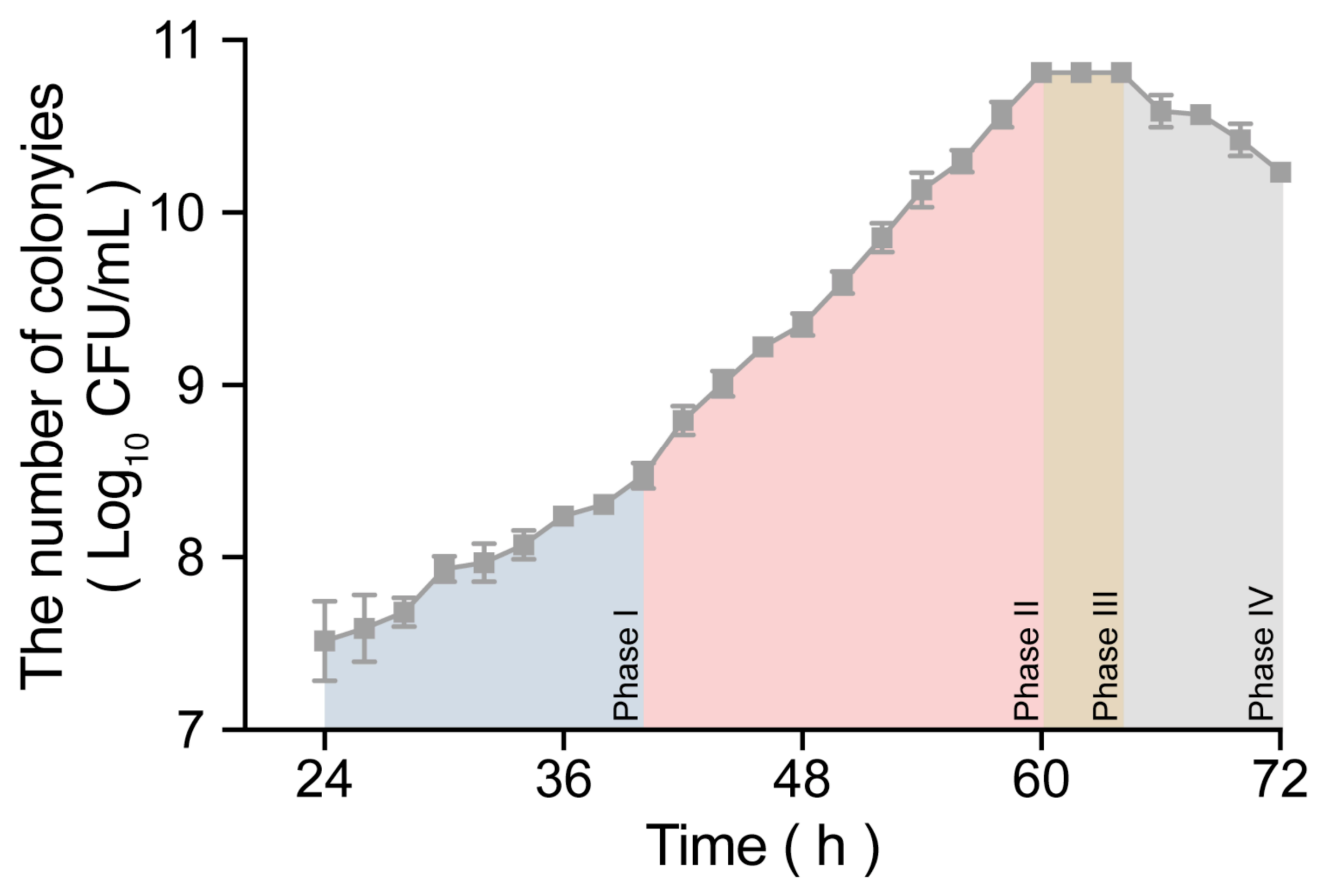
Growth curve of strain Bv-xms2024 determined by viable counts. Bv-xms2024 was cultured in LB broth at 25 °C with shaking at 170 rpm. Samples were collected every 2 h from 24 to 72 h, and viable cells were enumerated by serial dilution and plate counting. Data are plotted as log_10_(CFU/mL) and shown as mean ± SD (n = 3 independent biological replicates). The indicated phases were used for growth-kinetics analysis: Phase I, lag phase (24–40 h); Phase II, exponential growth phase (40–60 h); Phase III, stationary phase (60–64 h); and Phase IV, decline phase (64–72 h).

As shown in Figure 3, Bv-xms2024 exhibited a prolonged growth cycle under the tested culture conditions. The viable count remained relatively low before 40 h, corresponding to the lag phase. A rapid increase in viable cell density was observed from 40 to 60 h, indicating the exponential growth phase. During this period, the liquid culture gradually developed orange-red pigmentation, consistent with the accumulation of pigmented bacterial cells. The stationary phase occurred between 60 and 64 h, followed by a decline phase after 64 h.

Based on viable cell counts, the growth curve from 24 to 72 h was divided into four phases: lag phase (24–40 h), exponential growth phase (40–60 h), stationary phase (60–64 h), and decline phase (64–72 h). During the lag phase, the specific growth rate (μ) was 0.139 ± 0.023 h^−1^, corresponding to a generation time of 5.09 ± 0.83 h. During the exponential growth phase, μ increased to 0.2616 ± 0.0003 h^−1^, and the generation time decreased to 2.650 ± 0.003 h. The stationary phase showed a μ value close to zero, whereas the decline phase showed a negative growth slope of −0.1518 ± 0.0002 h^−1^, corresponding to a half-life of 4.565 ± 0.006 h.

### 3.5. Antibiotic Susceptibility Testing

Because species-specific CLSI interpretive criteria are not available for *Brevundimonas vesicularis*, the disk diffusion results were used only to describe the inhibition-zone profile of Bv-xms2024. As summarized in Table 4, no inhibition zones were observed for ampicillin, pipemidic acid, norfloxacin, sulfadiazine, and lincomycin. In contrast, measurable inhibition zones were observed for the remaining tested antibiotics, with doxycycline showing the largest inhibition zone (60 mm), followed by chloramphenicol (50 mm), gentamicin (45 mm), and kanamycin and tetracycline (44 mm each).

### 3.6. Carotenoid Production and Metabolomic Profiling

Using liquid chromatography-tandem mass spectrometry (LC–MS/MS), a quantitative analysis of 68 carotenoids in the metabolites of strain Bv-xms2024 was conducted. The results revealed that at 96 h, phytoene was the most abundant carotenoid among the bacterial metabolites, with a concentration of 420.42 ± 98.11 μg/g dry biomass (mean ± SD, n = 3 independent biological replicates). This was followed by β-carotene (21.66 ± 4.24 μg/g), astaxanthin (9.55 ± 1.78 μg/g), zeaxanthin (8.44 ± 1.73 μg/g), and phytofluene (5.01 ± 1.32 μg/g). These findings indicate that although Bv-xms2024 can synthesize downstream products like astaxanthin, a large amount of the precursor phytoene is retained.

### 3.7. Orthogonal Optimization of Cultivation Parameters for Phytoene Accumulation

**Figure 4:**
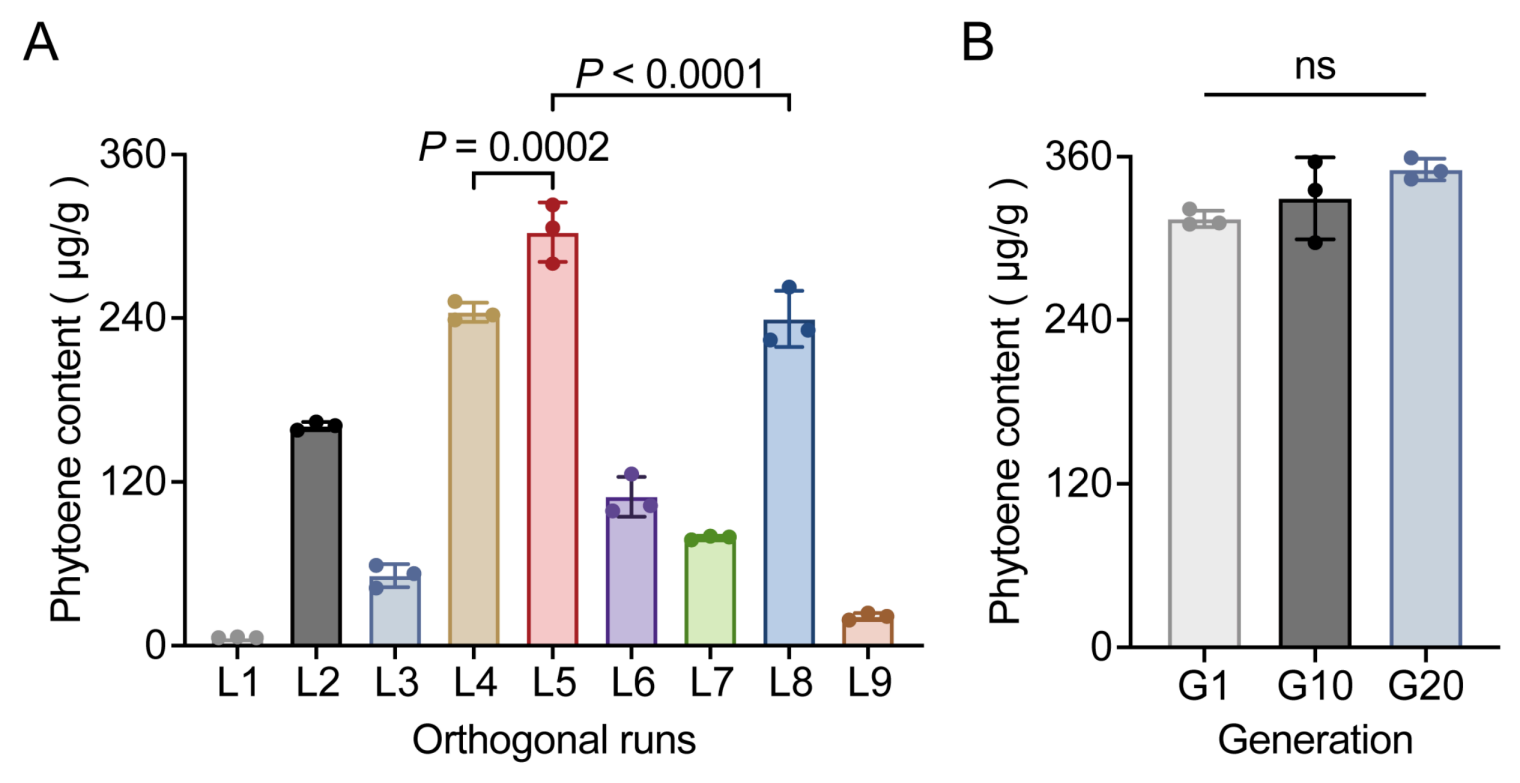
Orthogonal optimization of cultivation parameters and production stability for phytoene accumulation in Bv-xms2024. (**A**) Phytoene levels obtained from the L9 (3^3^) orthogonal runs. The conditions for each run were as follows: L1, 20 °C/48 h/pH 6.5; L2, 20 °C/72 h/pH 7.0; L3, 20 °C/96 h/pH 7.5; L4, 25 °C/72 h/pH 6.5; L5, 25 °C/96 h/pH 7.0; L6, 25 °C/48 h/pH 7.5; L7, 30 °C/96 h/pH 6.5; L8, 30 °C/48 h/pH 7.0; and L9, 30 °C/72 h/pH 7.5. (**B**) Phytoene levels at generations G1, G10, and G20 during serial passaging under the selected condition of 25 °C, initial pH 7.0, and 96 h. Bars represent mean ± SD (n = 3), and dots indicate individual biological replicates. Statistical analysis was performed using one-way ANOVA followed by Tukey’s multiple comparisons test; ns indicates no significant difference. Different colors are used only for visual distinction among the experimental groups and do not represent additional variables.

An L9 (3^3^) orthogonal array was used to evaluate the combined effects of cultivation time, temperature, and initial pH on phytoene accumulation in Bv-xms2024 (Table 1). Phytoene levels differed substantially among the nine runs (Figure 4A), ranging from 6.00 ± 0.33 μg/g in L1 (48 h, 20 °C, pH 6.5) to 303.24 ± 21.78 μg/g in L5 (96 h, 25 °C, pH 7.0) (mean ± SD, n = 3). The next highest levels were observed in L4 (244.31 ± 7.04 μg/g) and L8 (239.31 ± 20.56 μg/g). One-way ANOVA followed by Tukey’s multiple comparisons test showed that L5 produced significantly higher phytoene levels than L4 (*p* = 0.0002) and L8 (*p* < 0.0001). Main-effect range analysis based on level means indicated that initial pH had the greatest effect on phytoene accumulation, followed by temperature, whereas cultivation time showed a comparatively smaller effect within the tested range of 48–96 h. Accordingly, the best-performing condition was 96 h, 25 °C, and pH 7.0, corresponding to run L5.

To assess multi-generation production stability, phytoene levels were quantified at generations G1, G10, and G20 under the selected cultivation condition of 25 °C, initial pH 7.0, and 96 h (Figure 4B). Phytoene levels were comparable across generations: 314.62 ± 6.07 μg/g at G1, 329.61 ± 29.98 μg/g at G10, and 354.01 ± 13.39 μg/g at G20 (mean ± SD, n = 3). No significant difference was detected among generations by one-way ANOVA (*p* = 0.114).

### 3.8. Complete Genome Assembly and Annotation of Strain Bv-xms2024

**Figure 5:**
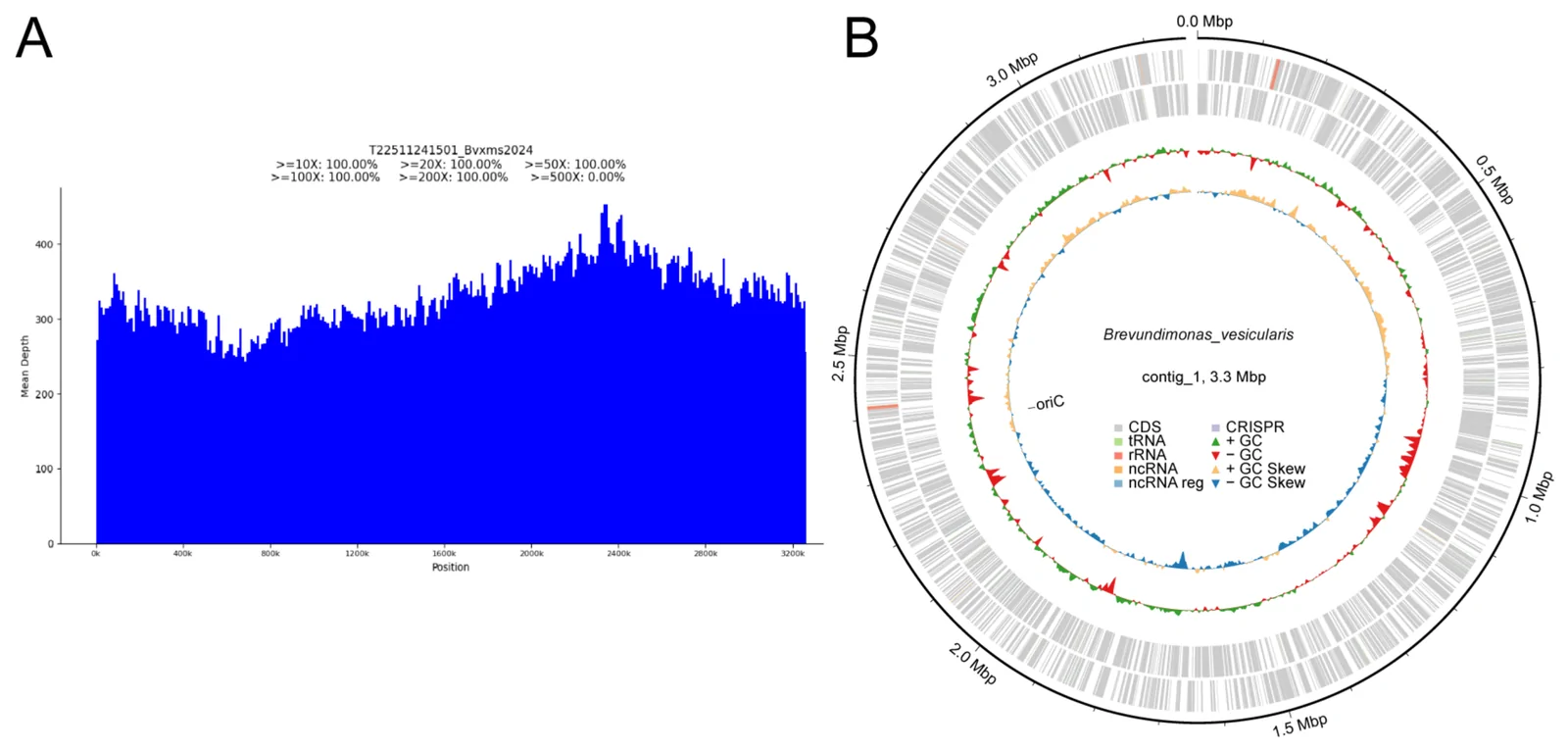
Genome assembly validation of strain Bv-xms2024 based on long-read sequencing. (**A**) Genome-wide sequencing depth distribution obtained by mapping clean long reads to the assembled genome. (**B**) Circular genome map of Bv-xms2024 showing genome structure and compositional variation, including GC content and GC skew.

Long-read sequencing generated 283,587 raw reads totaling 1,287,282,760 bases, with a raw-read N50 of 11,657 bp. After filtering, 178,130 clean reads totaling 1,193,912,872 bases were retained, with a clean-read N50 of 12,736 bp. De novo assembly produced a single contig of 3,263,986 bp, consistent with a complete genome assembly. Clean reads mapped back to the assembly with 100% genome coverage and a mean sequencing depth of 329.34×. The genome-wide depth distribution showed uniform coverage across the chromosome (Figure 5A). The assembled genome had a GC content of 66.05% and was identified as circular. A circular genome map was generated to summarize genome features and compositional variation, including GC content and GC skew (Figure 5B). Predicted genes were annotated using NR/Swiss-Prot, COG, KEGG, GO, and CAZy databases, and further screened against CARD, VFDB, and PHI. Homology-based screening against CARD identified 36 matches involving 23 genes, predominantly associated with antibiotic target alteration and antibiotic efflux. VFDB screening identified 160 homologous matches distributed across several functional categories, including immune modulation, motility, adherence, stress survival, biofilm formation, and effector delivery. PHI screening yielded 1027 gene–phenotype associations involving 653 genes, with reduced virulence and unaffected pathogenicity being the most frequently represented phenotype annotations.

### 3.9. Expression Analysis of Carotenoid Biosynthesis Genes

**Figure 6:**
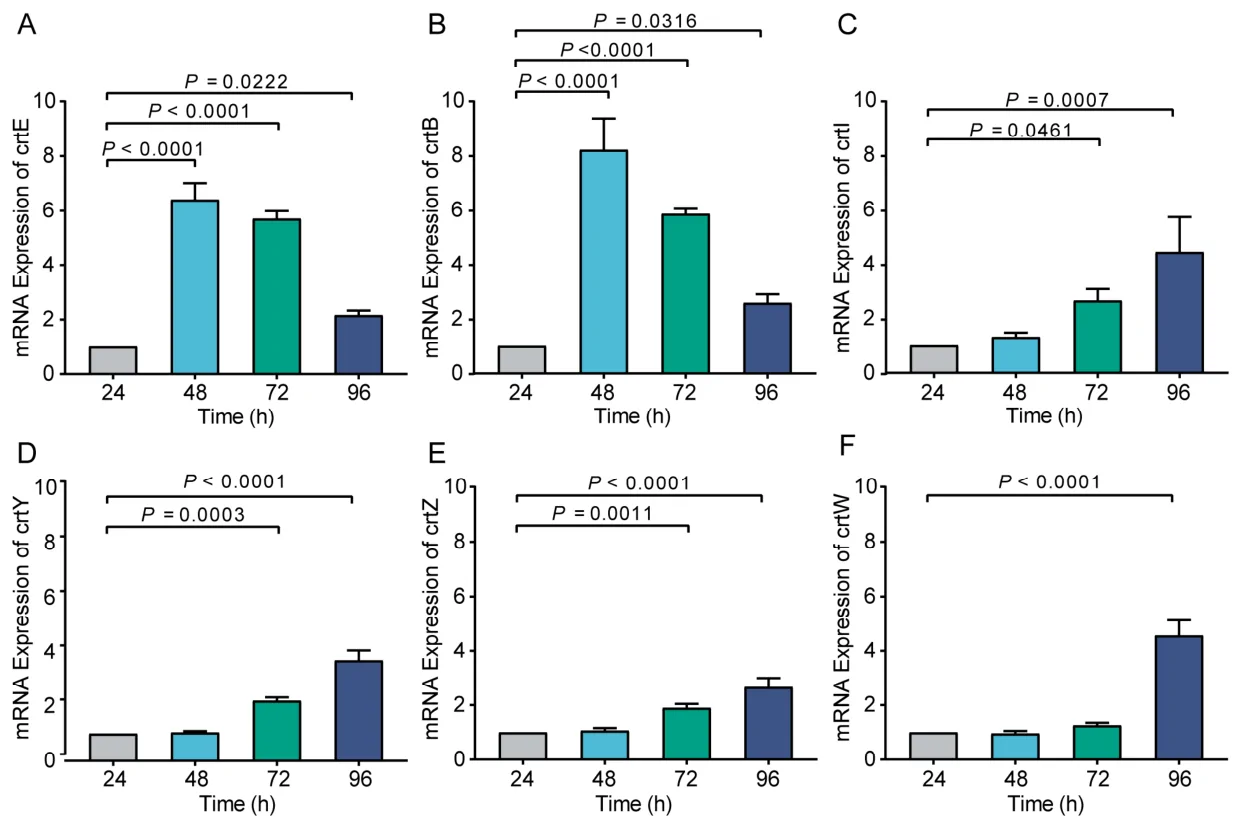
Expression levels of carotenoid biosynthetic genes in Bv-xms2024. Relative transcript levels of (**A**) *crtE*, (**B**) *crtB*, (**C**) *crtI*, (**D**) *crtY*, (**E**) *crtZ*, and (**F**) *crtW* were quantified by RT–qPCR at 24, 48, 72, and 96 h. Expression values were normalized to an internal reference gene and are presented relative to the 24 h group. Bars represent mean ± SD (n = 3 independent biological replicates). Statistical comparisons among time points were performed by one-way ANOVA with multiple comparisons; *p* values are shown above the brackets. Different colors indicate the four sampling time points: 24, 48, 72, and 96 h.

Carotenoid abundance was quantified by LC–MS/MS at 96 h, whereas RT-qPCR was performed at 24, 48, 72, and 96 h to track transcriptional changes in key carotenoid biosynthetic genes over time. As shown in Figure 6, the RT-qPCR results revealed significant changes in the expression of carotenoid biosynthetic genes over time.

Compared with 24 h, *crtE* and *crtB* were strongly upregulated at 48 h (*p* < 0.0001). This pattern is consistent with an early increase in the upstream module supplying precursors for phytoene formation. In contrast, *crtI*, *crtY*, *crtZ*, and *crtW* showed little change at 48 h, and no statistically significant differences were detected.

At 72 h, the expression of *crtE* and *crtB* remained significantly elevated (*p* < 0.0001), consistent with continued transcriptional activity of the upstream carotenoid biosynthetic module. Additionally, there was a slight but significant increase in the expression of *crtI*, *crtY*, and *crtZ* (*p* < 0.05), suggesting the initial activation of downstream genes in the carotenoid biosynthesis pathway. However, the expression of *crtW* continued to show no significant change compared with the 24 h control.

By 96 h, although the expression of *crtE* and *crtB* had decreased somewhat, they still exhibited an upregulated trend relative to the 24 h control (*p* < 0.05). Notably, the expression of *crtI*, *crtY*, *crtZ*, and *crtW* was significantly upregulated at this time point (*p* < 0.0001). At 96 h, increased expression of *crtI*, *crtY*, *crtZ*, and *crtW* coincided with the LC–MS/MS detection of downstream carotenoids, including astaxanthin. This pattern is consistent with stronger transcriptional activation of the downstream carotenoid biosynthetic module at 96 h.

### 3.10. Animal Safety Evaluation

**Figure 7:**
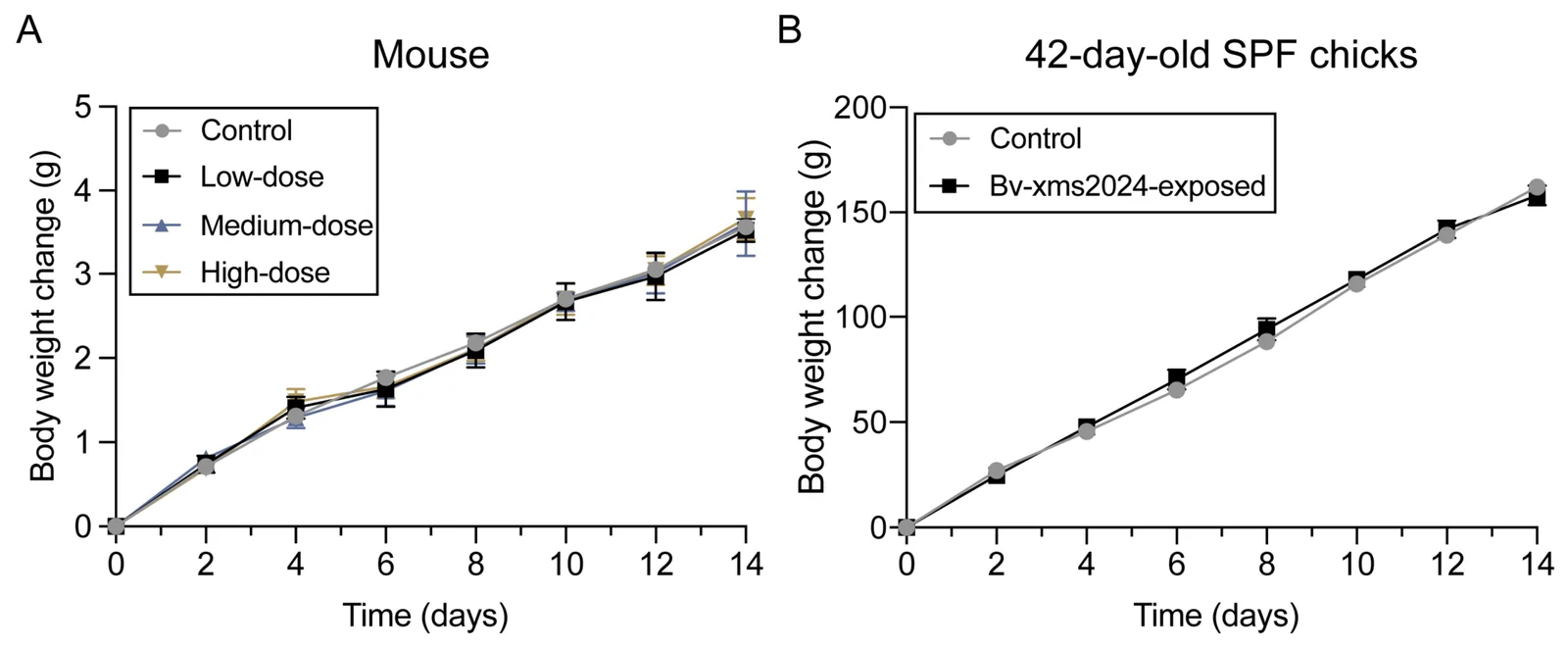
Body-weight changes in mice and SPF chicks during the 14-day experimental period. (**A**) Body-weight changes in mice in the control, low-dose, medium-dose, and high-dose groups. (**B**) Body-weight changes in 42-day-old SPF chicks in the control and Bv-xms2024-exposed groups. Data are presented as mean ± SD (n = 10 per group).

As shown in Figure 7, during the 14-day experimental period, all mice appeared healthy, exhibiting no adverse reactions or abnormal behaviors, such as ruffled fur or loss of appetite. There were no significant differences in weight gain between the control and low-, medium-, and high-dose Bv-xms2024 groups (*p* > 0.05). No mortality or morbidity was observed, and no significant differences in body-weight gain were detected between the control and Bv-xms2024-exposed groups, indicating no detectable short-term growth suppression or overt adverse effects under the tested conditions.

In the chicken experiments, both 42-day-old SPF chicks and 270-day-old laying hens showed no observable health problems throughout the 14-day study. No mortality or morbidity was recorded, and the birds maintained normal feeding behavior and activity levels. The control and Bv-xms2024-exposed SPF chicks showed similar body-weight gain trends, with no significant difference between groups (*p* > 0.05; Figure 7). Because body weight in laying hens may fluctuate with egg production cycles, their body-weight change data are not presented. The numerical body-weight data for mice and SPF chicks are provided in Appendix A (Table A1).

## 4. Discussion

In this study, we isolated and characterized a phytoene-producing *Brevundimonas vesicularis* strain (Bv-xms2024). Under the tested heterotrophic cultivation conditions, phytoene reached 420.42 ± 98.11 μg/g dry biomass at 96 h and remained substantially higher than other detected carotenoids, including β-carotene (21.66 ± 4.24 μg/g), astaxanthin (9.55 ± 1.78 μg/g), and zeaxanthin (8.44 ± 1.73 μg/g). The dominant phytoene profile is the main feature of this isolate. A direct ranking against the literature requires caution because phytoene values are reported on different bases (e.g., mg/L, mg/g dry biomass, or pellet/tissue basis) and are obtained under different cultivation regimes. Reported higher titers are often achieved by engineering or by reducing downstream conversion, for example, inhibitor-guided redirection in *Blakeslea trispora* (5.02 mg/g dry biomass) [9] and phytoene desaturase downregulation in *Dunaliella salina* (up to 1.08 mg/g dry cell weight) [23]. These approaches add process steps and may raise regulatory and cost considerations. In contrast, Bv-xms2024 shows phytoene dominance without genetic modification or pathway inhibition, providing a practical baseline for further optimization (Table 5). This optimization focuses on core physical parameters (time, temperature, and pH) and defines a workable process window for Bv-xms2024 under flask-scale cultivation. It does not represent an upper limit. In addition, phytoene production was stable during serial passaging, with comparable titers at G1, G10, and G20 under the selected condition (Figure 4B; one-way ANOVA, *p* = 0.114). While this supports basic production robustness at the flask scale, longer-term passaging and continuous-culture validation will be needed to fully establish industrial stability. Further gains in titer and productivity will likely come from variables that directly affect biomass formation and pathway flux, including carbon/nitrogen supply, oxygen transfer (agitation and aeration), inoculum size, and scale-up under controlled bioreactor operation.

**Figure 8:**
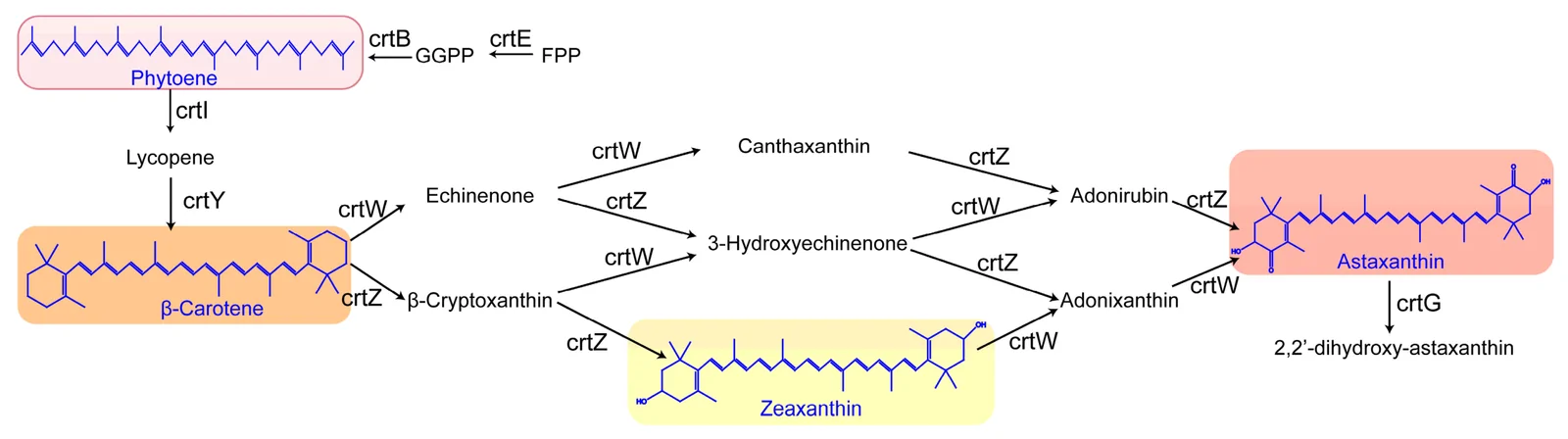
Proposed Carotenoid Biosynthetic Pathway in Bv-xms2024. Note: This working model is inferred from gene annotation, time-dependent RT-qPCR patterns, and LC–MS/MS detection of representative carotenoids at 96 h, with reference to carotenoid biosynthetic gene clusters characterized in other *Brevundimonas* strains [14,15,26].

Gene expression analysis showed early upregulation of *crtE* and *crtB* at 48 h, whereas increased expression of downstream genes (*crtI*, *crtY*, *crtZ*, and *crtW*) was observed at 96 h, coinciding with the detection of downstream carotenoids such as astaxanthin (Figure 8). Similar carotenoid biosynthetic gene clusters involving these key pathway genes have been characterized in other *Brevundimonas* strains [14,15,26]. The substantially higher abundance of phytoene relative to β-carotene, astaxanthin, and zeaxanthin may reflect limited net conversion of phytoene to downstream carotenoids under the tested conditions. However, the present transcript and 96-h metabolite data do not identify a specific rate-limiting step or directly define metabolic flux. Future studies combining targeted enzyme assays with time-resolved carotenoid profiling may further clarify the regulation of carotenoid accumulation in Bv-xms2024.

Disk-diffusion testing revealed substantial variation in inhibition-zone diameters among the tested antibiotics. No inhibition zones were observed for ampicillin, pipemidic acid, norfloxacin, sulfadiazine, and lincomycin, whereas doxycycline produced the largest inhibition zone (60 mm). Because species-specific CLSI interpretive criteria are unavailable for *B. vesicularis*, these results are interpreted descriptively. From a process-development perspective, further evaluation of Bv-xms2024 will require optimization of cultivation and downstream processing, together with validated product analysis and comprehensive strain-safety assessment.

Some strains of *Brevundimonas vesicularis* have been reported as opportunistic pathogens associated with infections including peritonitis, bacteremia, pulmonary infection, and liver abscess [27,28,29,30,31]. In the present study, no mortality, morbidity, overt clinical abnormalities, or significant impairment of body-weight gain was observed in mice or chickens during the 14-day observation period under the tested conditions, providing initial evidence of good short-term tolerance of Bv-xms2024. Hematological, biochemical, and pathological assessments were not included in the present study; these endpoints, together with longer-term observations, can be incorporated into future studies to further characterize strain-level safety.

## 5. Conclusions

Bv-xms2024 was identified as a naturally occurring *Brevundimonas vesicularis* strain with marked phytoene accumulation. The strain predominantly accumulated phytoene after 96 h of cultivation, showed time-dependent expression of carotenoid biosynthetic genes, and maintained comparable phytoene levels during serial passaging under the selected cultivation condition. Short-term exposure in mice and chickens caused no observable adverse effects under the tested conditions. These findings support Bv-xms2024 as a natural microbial resource for phytoene production, although further process optimization, purification validation, and safety assessment are required for food- and feed-related applications.

## Figures and Tables

**Table 1:** L9 (3^3^) orthogonal array showing experimental runs and factor settings.

Run	Temperature (°C)	Time (h)	pH
1	20	48	6.5
2	20	72	7.0
3	20	96	7.5
4	25	72	6.5
5	25	96	7.0
6	25	48	7.5
7	30	96	6.5
8	30	48	7.0
9	30	72	7.5

**Table 2:** Primer sequences used for RT-qPCR analysis.

Genes	Sequence (5′–3′)	Product Length (bp)	GenBank Number
*c* *rtY*	F: GCTGGGTCATAGAGACGGTT	189	ABC50115.1
	R: GCCGGTAGTCGATGAAACA		
*c* *rtW*	F: CAGGTGCCGAAGGTGAGAG	205	ABC50116.1
	R: CTTTCGCACTATTTCGGCTG		
*c* *rtI*	F: TCGGTCTATGACAAGGTCGC	235	ABC50114.1
	R: AGGGCTAGATCGATGAGGT		
*c* *rtB*	F: CGGTCTTCACCGCCTTTCAG	143	ABC50113.1
	R: GATAGGCGTATCCAGCGTG		
*c* *rtE*	F: TCCATCAGGTCGTCGCAAAG	203	ABC50110.1
	R: CTGCGTCGGATCAACGATCT		
*c* *rtZ*	F: CACCCACAGAAGCCGAAGG	186	ABC50108.1
	R: ACGGCCTATGGGATGGTCT		

**Table 3:** Physiological and biochemical characteristics of strain Bv-xms2024.

Test	Result
Gram staining	Negative
Cell morphology	Short rods
Catalase test	Positive
Oxidase test	Negative
Citrate utilization	Positive
Indole production	Negative
Urease activity	Negative
Hydrogen sulfide production	Negative
Glucose fermentation	Positive
Sucrose fermentation	Positive
Lactose fermentation	Negative

**Table 4:** Antibiotic disk diffusion profile of strain Bv-xms2024.

Antibiotic Class	Antibiotic	Disk Content	Inhibition Zone Diameter (mm)
β-Lactams	Ampicillin	10 μg	0
	Penicillin	10 U	19
	Amoxicillin	20 μg	32
	Cephalexin	30 μg	39
	Cefaclor	30 μg	34
	Ceftazidime	30 μg	12
	Cefepime	30 μg	20
Aminoglycosides	Kanamycin	30 μg	44
	Spectinomycin	100 μg	31
	Gentamicin	10 μg	45
Quinolones	Pipemidic acid	20 μg	0
	Norfloxacin	10 μg	0
	Ciprofloxacin	5 μg	25
	Moxifloxacin	5 μg	32
Macrolides	Azithromycin	15 μg	38
	Erythromycin	15 μg	42
Tetracyclines	Doxycycline	30 μg	60
	Tetracycline	30 μg	44
Sulfonamides	Sulfamethoxazole	23.75 μg	35
	Sulfadiazine	300 μg	0
Lincosamides	Lincomycin	2 μg	0
Polypeptides	Polymyxin B	300 U	21
Phenicols	Chloramphenicol	30 μg	50

**Table 5:** Comparison of phytoene production in Bv-xms2024 and representative naturally carotenogenic microorganisms reported in the literature.

Organism (Type)	Cultivation Mode/Key Conditions	Reported Phytoene Level (as Reported)	Strategy/Context for Phytoene Accumulation	Reference
*B. vesicularis* Bv-xms2024 (bacterium)	Heterotrophic, LB, 25 °C, 170 rpm, 96 h	420.42 ± 98.11 µg/g (dry biomass)	No genetic modification; no pathway inhibitor; phytoene-dominant profile under tested conditions	This study
*D. salina* DF15 (microalga)	25 °C, pH 7.5, mixing 100 rpm; light 200 µmol photons m^−2^ s^−1^	1.4–2.2 mg/L (0.21–0.36% AFDW)	Light-dependent carotenoid production; phytoene detectable under defined wavelengths	Sui et al., 2021 [24]
*Chlorococcum* sp. UTEX B 3056 (microalga)	With fluridone post-incubation	~33 µg/mg cell tissue (control: non-detectable)	Reducing phytoene desaturase activity chemically to limit downstream conversion	Laje et al., 2019 [25]
*Blakeslea trispora* (fungus)	Mated cultures; inhibitors + process optimization	5.02 mg/g dry biomass; 203.91 mg/L	Metabolic flux redirected by carotenoid biosynthesis inhibitors	Mantzouridou et al., 2024 [9]

Note: AFDW, ash-free dry weight. Normalization bases differ across studies (e.g., dry biomass, AFDW, tissue basis, or wet-pellet basis); therefore, values are intended for qualitative benchmarking rather than direct yield ranking.

## Data Availability

Public access to portions of the complete dataset is restricted because they contain proprietary technical information associated with patent commercialization.

## Supplementary Materials

The following supporting information can be downloaded at: https://www.mdpi.com/article/10.3390/foods15172981/s1, Supplementary Method S1: Detailed Sample Preparation and UPLC–APCI–MS/MS Analysis of Carotenoids; Table S1: MRM transitions used for the targeted LC–MS/MS analysis of the major carotenoids reported in this study.

## Abbreviations

The following abbreviations are used in this manuscript:

ANOVA	Analysis of variance
AFDW	Ash-free dry weight
bp	Base pair
CAZy	Carbohydrate-Active enZymes database
CARD	Comprehensive Antibiotic Resistance Database
CFU	Colony-forming unit
CLSI	Clinical and Laboratory Standards Institute
COG	Clusters of Orthologous Groups
GO	Gene Ontology
KEGG	Kyoto Encyclopedia of Genes and Genomes
LB	Luria–Bertani
LC–MS/MS	Liquid chromatography–tandem mass spectrometry
NCBI	National Center for Biotechnology Information
NR	Non-redundant protein database
OD_600_	Optical density at 600 nm
PBS	Phosphate-buffered saline
PHI	Pathogen–Host Interactions database
RT-qPCR	Reverse transcription–quantitative polymerase chain reaction
SD	Standard deviation
SPF	Specific pathogen-free
VFDB	Virulence Factor Database

## Appendix A

**Table A1:** Body-weight changes in mice and SPF chicks during the 14-day experimental period.

Group	Mouse				42-Day-Old SPF Chicks	
Time (d)	Control group (g)	Low-dose group (g)	Medium-dose Group (g)	High-dose Group (g)	Control Group (g)	Bv-xms2024-exposed Group (g)
0	0.00 ± 0.00	0.00 ± 0.00	0.00 ± 0.00	0.00 ± 0.00	0.00 ± 0.00	0.00 ± 0.00
2	0.71 ± 0.11	0.74 ± 0.10	0.81 ± 0.07	0.69 ± 0.08	27.0 ± 4.78	24.7 ± 3.22
4	1.31 ± 0.22	1.41 ± 0.13	1.29 ± 0.12	1.48 ± 0.15	45.6 ± 5.01	47.7 ± 3.85
6	1.77 ± 0.15	1.63 ± 0.21	1.61 ± 0.18	1.66 ± 0.14	65.2 ± 5.23	70.2 ± 4.70
8	2.18 ± 0.19	2.09 ± 0.20	2.10 ± 0.17	2.11 ± 0.14	88.3 ± 6.91	94.1 ± 5.13
10	2.70 ± 0.13	2.67 ± 0.22	2.67 ± 0.11	2.70 ± 0.19	115.5 ± 4.18	117.8 ± 3.63
12	3.05 ± 0.28	2.97 ± 0.28	3.01 ± 0.24	3.04 ± 0.17	138.8 ± 4.09	141.6 ± 4.18
14	3.56 ± 0.17	3.52 ± 0.14	3.60 ± 0.39	3.66 ± 0.25	162.0 ± 4.52	158.1 ± 4.67

Note: values are presented as mean ± SD (n = 10 per group).
