# Supplementary material for: Isolation and Characterization of a Naturally Occurring Brevundimonas vesicularis Strain Exhibiting High Phytoene Accumulation
Source: Foods. 2026 Aug 25;15(17):2981. doi: 10.3390/foods15172981 (PMC13564605; doi:10.3390/foods15172981)
Supplement: Supplementary file 1 [file foods-15-02981-s001.zip › foods-4502630-supplementary.pdf]

Supplementary Materials

Isolation and Characterization of a Naturally Occurring *Brevundimonas vesicularis* Strain Exhibiting High Phytoene Accumulation

Supplementary Method S1. Detailed Sample Preparation and UPLC–APCI–MS/MS Analysis of Carotenoids

Analytical standards were obtained from Sigma-Aldrich (St. Louis, MO, USA) and BOC (New York, NY, USA). Stock standard solutions were prepared at 1 mg/mL in methyl tert-butyl ether (MTBE)/methanol and stored at −20 °C until use.

Freeze-dried cell-pellet samples were ground into a fine powder using a ball mill at 30 Hz for 1 min. A 50 mg aliquot of the freeze-dried powder was extracted with 0.5 mL of n-hexane/acetone/ethanol (1:1:1, *v*/*v*/*v*) containing 0.01% butylated hydroxytoluene (BHT). The mixture was vortexed for 10 min at room temperature and sonicated in an ice-water bath for 5 min. After centrifugation at 12,000 rpm for 5 min at 4 °C, the supernatant was collected. The remaining residue was extracted once more under the same conditions, and the two supernatants were combined. The combined extract was concentrated to dryness and reconstituted in 150 μL of dichloromethane. The reconstituted solution was filtered through a 0.22 μm membrane and transferred to an amber vial for LC–MS/MS analysis. The extraction procedure was performed under light-protected conditions to minimize carotenoid oxidation and degradation.

Carotenoid analysis was performed using an ExionLC™ AD ultra-performance liquid chromatography system coupled to a QTRAP® 6500+ mass spectrometer (SCIEX, Framingham, MA, USA). Chromatographic separation was achieved on a YMC C30 column (YMC Co., Ltd., Kyoto, Japan) (3 μm, 100 mm × 2.0 mm i.d.). Mobile phase A consisted of methanol/acetonitrile (1:3, *v*/*v*) containing 0.01% BHT and 0.1% formic acid, whereas mobile phase B consisted of MTBE containing 0.01% BHT. The gradient program was as follows: 0–3 min, 0% B; 3–5 min, a linear increase from 0% to 70% B; 5–9 min, a linear increase from 70% to 95% B; 9–10 min, a return from 95% to 0% B; and 10–11 min, 0% B. The flow rate was 0.8 mL/min, the column temperature was maintained at 28 °C, and the injection volume was 2 μL.

The mass spectrometer was equipped with an atmospheric pressure chemical ionization source and operated in positive-ion mode (APCI+). The source temperature was set at 350 °C and the curtain gas pressure was 25 psi. Carotenoids were analyzed using scheduled multiple reaction monitoring (MRM). Analyte-specific precursor/product ion transitions (Q1/Q3) were used for targeted detection. Declustering potential (DP) and collision energy (CE) were optimized for individual MRM transitions, and the transitions monitored during each acquisition period were selected according to the retention window of the corresponding analytes. The Q1/Q3 transitions for the major carotenoids reported in this study are provided in Supplementary Table S1. Data were acquired using Analyst 1.6.3 software and quantitative data were processed using MultiQuant 3.0.3 software (SCIEX).

Qualitative identification was performed using the MetWare Database (MWDB), which was constructed based on analytical standards, together with chromatographic retention characteristics and analyte-specific Q1/Q3 transitions under scheduled MRM acquisition. Chromatographic peaks were integrated using MultiQuant 3.0.3. Quantification was performed using analyte-specific external-standard calibration curves. Carotenoid standard solutions were prepared at 0.001, 0.005, 0.01, 0.05, 0.1, 0.5, 1, 5, 10, and 25 μg/mL, with standard concentration as the independent variable and chromatographic peak area as the response variable.

Carotenoid contents in freeze-dried cell samples were calculated according to the equation $C = c \times V/(1000 \times m)$, where C is the carotenoid content in the sample (μg/g dry biomass), c is the analyte concentration obtained from the corresponding calibration curve (μg/mL), V is the reconstitution volume (μL), and m is the mass of the freeze-dried sample used for extraction (g). Accordingly, all carotenoid contents expressed as μg/g in this study were normalized to dry biomass.

Table S1. MRM transitions used for the targeted LC–MS/MS analysis of the major carotenoids reported in this study.

Compound	Q1 (Da)	Q3 (Da)	Ion Mode
(E/Z)-Phytoene	545.3	121.2	Positive
Phytofluene	543.5	81.2	Positive
β-Carotene	537.6	177.1	Positive
Astaxanthin	597.3	147.1	Positive
Zeaxanthin	569.4	477.5	Positive
